# Development and Field Construction Protection of a Fiber Bragg Grating-Geogrid Integrated System in Asphalt Pavements

**DOI:** 10.3390/ma19102115

**Published:** 2026-05-18

**Authors:** Hui Wang, Da Zhang, Qiaoyi Li, Guangqing Yang, Peng Xu, Xunmei Liang

**Affiliations:** 1 Cangzhou Qugang Expressway Construction Co., Ltd., Cangzhou 061000, China; czqg134@163.com; 2 School of Traffic and Transportation, Shijiazhuang Tiedao University, Shijiazhuang 050043, China; 3 School of Urban Geology and Engineering, Hebei GEO University, Shijiazhuang 052161, China; 4 School of Civil Engineering, Shijiazhuang Tiedao University, Shijiazhuang 050043, China; yanggq@stdu.edu.cn; 5 State Key Laboratory of Mechanical Behavior and System Safety of Traffic Engineering Structures, Shijiazhuang Tiedao University, Shijiazhuang 050043, China; sdxplt@163.com; 6 Shandong Road New Materials Co., Ltd., Taian 271000, China; ludelxm@163.com

**Keywords:** fiber Bragg grating, geogrid, asphalt pavement, full-scale field test, construction damage

## Abstract

Facing the challenges in field monitoring of the mechanical response of geogrids in asphalt pavements, this study integrated two types of Fiber Bragg Grating (FBG) sensors, unarmored and armored, into geogrids using the pillar-stitching technique on industrial warp-knitting production lines. The integrated FBG-geogrid systems were comprehensively evaluated in both wound and flattened configurations, enabling the selection of a sensor type suitable for industrial production. After precise strain calibration, a full-scale field damage test was performed during the construction of the Qu-Gang Expressway in Hebei Province, China. The results demonstrate that the helical steel armor layer significantly enhances the mechanical durability of the FBG sensor. Specifically, the armored sensor maintained stable optical transmission over its entire 60-m length, with an average performance retention rate of 98.86% in the flattened state. Moreover, a strong linear correlation was established between the wavelength shift of the armored FBG sensor and the tensile strain of the geogrids. In contrast, the unarmored FBG sensor underwent irreversible shear deformation during production and contained at least two breakpoints. Additionally, a protection scheme employing fiberglass-reinforced silicone rubber on the hot side and standard silicone rubber on the cold side effectively shielded the sensors from high-temperature and compaction loads during asphalt paving. Consequently, the proposed FBG-geogrid integration method and the corresponding field protection strategy provide technical support for the real-time monitoring of geogrid performance in asphalt pavements and have significant engineering value.

## 1. Introduction

Warp-knitted geogrids are widely embedded within asphalt pavement layers to extend service life [1,2]. Extensive research confirms that the lateral confinement and structural reinforcement provided by these geosynthetic interlayers significantly enhance mechanical performance, particularly regarding resistance to cracking, rutting, and fatigue [3,4,5,6,7]. However, achieving direct, accurate, and real-time monitoring of the mechanical response of geogrids within asphalt pavements remains challenging. While conventional resistive sensors are suitable for controlled laboratory scenarios [8], their survivability, long-term stability, and measurement accuracy often prove inadequate for full-scale field applications. This limitation stems primarily from harsh field conditions, characterized by significant mechanical stress and high-temperature loading during construction [9].

In recent years, Fiber Bragg Grating (FBG) technology has been increasingly used for mechanical monitoring of geogrids, owing to its advantages, such as immunity to electromagnetic interference, distributed monitoring capability, long-term stability, and high precision [10,11]. Currently, methods for integrating FBG sensors with geogrids fall into two main categories: external attachment and embedded installation. External attachment refers to affixing FBG sensors onto the geogrid surface using epoxy resin adhesives [12,13,14,15] or specialized fixtures [16]. However, the quality of the manually operated cementation layer is the core of the strain transfer between the FBG sensor and the measured geosynthetics. Existing research indicates that adhesive layers not only have the risk of debonding and damage [17], but also tend to induce a shear lag effect between FBG sensors and geogrids [18]. This effect is mainly governed by parameters of the adhesive layers, such as bonding length, thickness, width, and Young’s modulus. Meanwhile, the manual application of adhesive materials leads to discreteness of the parameters of the adhesive layers [19]. This results in non-negligible and random errors between the strain measurements from FBG sensors and actual strain in geogrids. This inherently compromises the reliability of measurements. Embedded installation means that FBG sensors are integrated with geosynthetics through weaving and stitch-bonding. The development of an intelligent monitoring system capable of detecting the structural failure of hydraulic engineering structures was started by the German Federal Ministry of Education and Research to extreme flood events. The idea is to integrate optical fiber sensors into geosynthetics to monitor the stability of dikes [20]. A novel approach for embedding of optical fibers in laminates is integration in the reinforcement fabric, which was discussed by Schuster et al. [21], Koncar [22] and Harlin et al. [23] for different types of optical fibers woven into textile structures. In addition, multifunctional geotextile produced by Alpe Adria Textil, Italy, was fabricated by integrating an optical fiber arranged along the warp direction into the warp-knitted geogrid [24].

However, existing studies on embedded installation mostly focus on the integration of optical fiber and geosynthetics through weaving and stitch-bonding, and its application in geotechnical engineering such as dams, slopes and embankments. To date, research on the application of FBG-geogrid integrated systems in reinforced highway asphalt pavements remains limited. Within the engineering context of geosynthetic-reinforced asphalt surface layers, the combined effects of manufacturing processes, roller compaction loads, and aggregate-induced shear stresses on the integrated system are not yet fully understood. Notably, these construction and service conditions readily induce FBG bending, resulting in FBG power attenuation. Such signal loss is highly undesirable, as it directly degrades measurement fidelity and compromises the reliability of long-term structural health monitoring systems.

To address these limitations, this study develops an integrated FBG-geogrid system utilizing an industrial warp-knitting production technique and proposes a corresponding implementation methodology for field applications. Specifically, 60-m-long helical steel-armored and unarmored FBG-geogrids were fabricated using warp-knitted geogrids as the host matrix. Based on the optical signal transmission performance of both types under wound and flattened conditions, the FBG sensor configuration most suitable for industrial production was selected. Subsequently, the strain sensitivity coefficient was calibrated via laboratory uniaxial tensile tests. Finally, protective materials were optimized to prevent sensor damage during construction, validated through full-scale field damage tests conducted on the Qu-Gang Expressway project in Hebei Province, China.

## 2. Development of the FBG-Geogrid Integrated System

### 2.1. Materials

#### 2.1.1. Ultra-Weak FBG Sensors

Ultra-weak Fiber Bragg Gratings (FBGs) are fabricated using time-division multiplexing (TDM) technology [25]. This technique allows for the inscription of thousands of gratings with identical periods on a single optical fiber, enabling high-precision temperature and strain measurements through wavelength modulation and optical frequency domain reflectometry (OFDR)-based positioning. Their measurement principle is analogous to that of conventional FBGs [26]. Compared with the traditional FBG which is suitable for localized monitoring at critical structural points, featuring densely spaced grating points, ultra-weak FBGs support distributed, continuous monitoring and are particularly advantageous for long-distance sensing applications.

**Figure 1:**
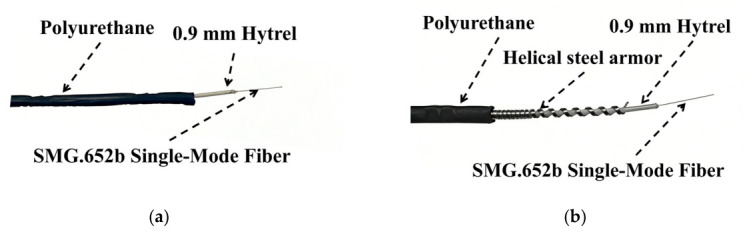
Physical structure of ultra-weak FBG sensors: (**a**) The unarmored sensor; (**b**) The armored sensor.

**Figure 2:**
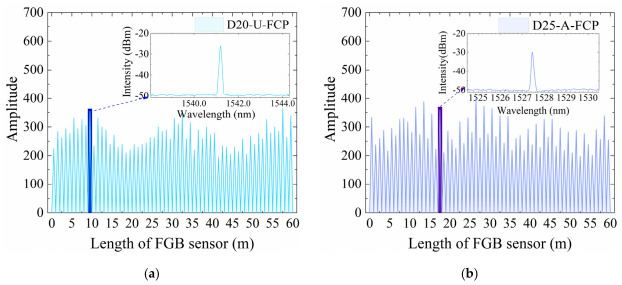
Initial optical signal performance of FBG sensors: (**a**) The unarmored sensor; (**b**) The armored sensor.

Two types of commercial ultra-weak FBG sensors—unarmored and helical steel armored—were employed in this study to fabricate the FBG-geogrid, as shown in Figure 1. The unarmored sensor has a diameter of 2.0 mm, a wavelength range from 1528 to 1568 nm, and a sensing point spacing of 1 m. The armored sensor has a diameter of 2.5 mm, the same wavelength range (from 1528 to 1568 nm), and the same 1-m point spacing. Prior to integration with the geogrid, the working wavelength and baseline optical signal transmission performance of both FBG sensors were evaluated according to the Test Method for Parameters of Distributed Optical Fiber Strain Measuring System (GB/T 43256-2023) [27]. The results are presented in Figure 2.

As shown in Figure 2, all 60 sensing points were detected, indicating an absence of optical signal interruption. The waveforms of the sensing points are distinct, showing stable and uniform optical signal transmission. These results verify the high signal quality and dependable sensing capabilities of the FBG sensors utilized in this research.

Note: In the figures, legend labels are assigned based on the sensor’s outer diameter, armoring status, and physical condition, using the format: Diameter (D20/D25)-Armored/Unarmored (A/U)-Physical Condition. The physical conditions are as commercially packaged (FCP), integrated into the geogrid in the wound condition (FWC), and integrated into the geogrid in the flattened condition (FFC). For example, an integrated system containing a 2.0-mm unarmored FBG in the wound condition is labeled D20-U-FWC.

#### 2.1.2. Glass-Fiber Geogrid

**Figure 3:**
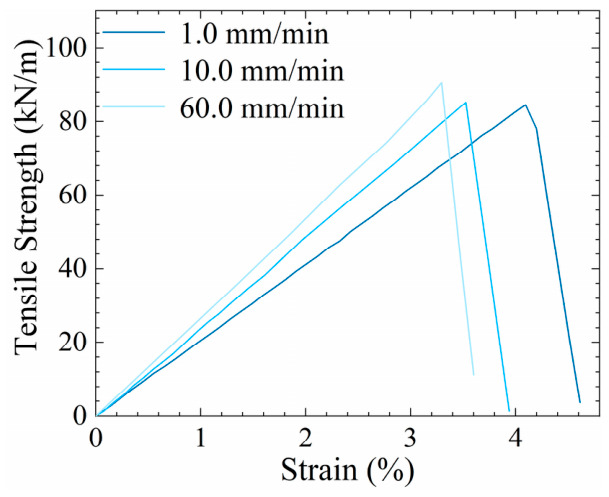
Stress–strain curves of glass-fiber geogrid.

A glass-fiber geogrid was selected as the host material for integrating the FBG sensors. In accordance with ASTM D6637 (Standard Test Method for Determining Tensile Properties of Geogrids by the Single or Multi-Rib Tensile Method) [28], single ribs of the glass-fiber geogrid were subjected to tensile tests at various extension rates. The results are presented in Figure 3.

### 2.2. Manufacturing Process

**Figure 4:**
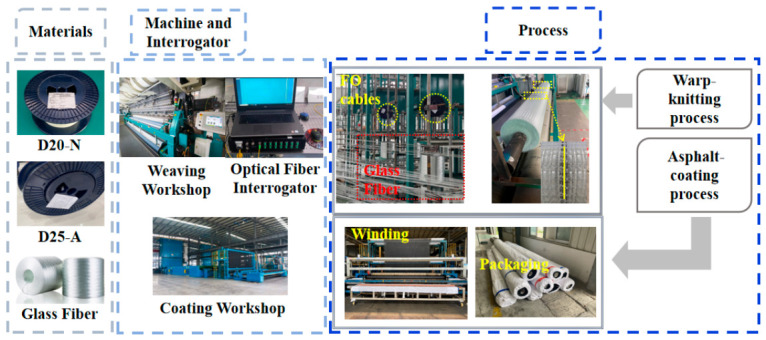
Complete manufacturing process of FBG-geogrids.

The complete manufacturing process for the FBG-geogrids is illustrated in Figure 4. The procedure encompasses raw material preparation, machine calibration, optical interrogator setup, warp knitting, and final asphalt coating. During the warp-knitting stage, the FBG sensors were fed synchronously into the knitting machine alongside the warp yarns. Physical integration was achieved through pillar stitching, which entangles the sensors with the geogrid structure. In the subsequent coating stage, modified asphalt was uniformly applied to the surface of the FBG-geogrid via an impregnation method. Finally, the coated FBG-geogrid was stretched in both the transverse and longitudinal directions after drying to establish its final structural geometry.

### 2.3. Sensing Performance of FBG-Geogrids

To select the FBG sensor type suitable for the industrial production of FBG-geogrids, the working performance of both sensors under wound and flattened conditions was evaluated based on the optical signal intensity of all sensing points. Furthermore, by incorporating the baseline optical signal performance of the two FBG sensors presented in Section 2.1.1, a performance retention rate (χ) and the average retention rate ($\bar{\chi }$) were introduced to quantify their working performance. The rates χ and $\bar{\chi }$ are defined as follows:

(1) $$\chi_{\mathrm{FWC}}=\frac{\xi_{\mathrm{FWC}}}{\xi_{\mathrm{FCP}}}\times 100\%,$$

(2) $$\chi_{\mathrm{FFC}}=\frac{\xi_{\mathrm{FFC}}}{\xi_{\mathrm{FCP}}}\times 100\%,$$

(3) $$\bar{\chi }=\frac{\sum \chi }{n}$$

where $\chi_{\mathrm{FWC}}$ and $\chi_{\mathrm{FFC}}$ represent the performance retention rates under the wound and flattened conditions, respectively; $\bar{\chi }$ is the average value of performance retention rate $\chi_{\mathrm{FWC}}$ and $\chi_{\mathrm{FFC}}$ in the certain length; $\xi_{\mathrm{FCP}}$ is the optical signal intensity of the sensing points in the commercially packaged (as-received) condition; and $\xi_{\mathrm{FWC}}$ and $\xi_{\mathrm{FFC}}$ are the optical signal intensities under the wound and flattened conditions after integration, respectively.

#### 2.3.1. Performance Under the Wound Condition

**Figure 5:**
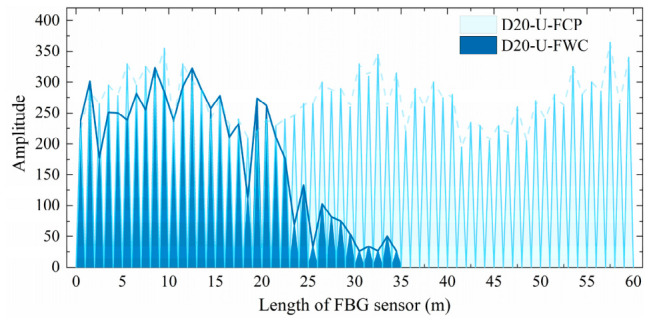
Initial optical signal performance of unarmored FBG sensors.

**Figure 6:**
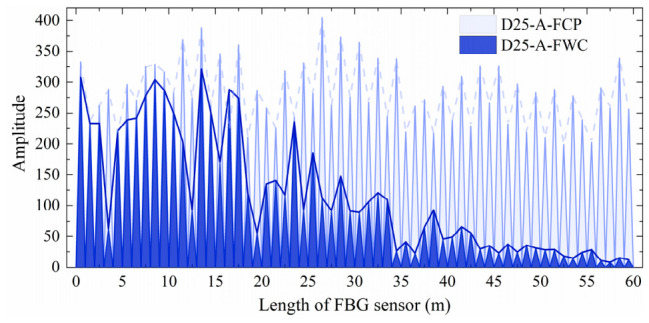
Initial optical signal performance of armored FBG sensors.

Figure 5 and Figure 6 compare the optical signal transmission performance of the commercially packaged FBG sensors with their geogrid-integrated counterparts in the wound condition. As shown in Figure 5 and Figure 6, the signals for D20-U-FCP and D25-A-FCP exhibit periodic fluctuations along their length in the as-received state. However, the two sensors show distinct attenuation characteristics after integration. The optical signal intensity of D20-U-FWC decreases slightly from 0 to 20 m, undergoes steep attenuation between 20 and 24 m, and disappears completely beyond 35 m. In contrast, D25-A-FWC shows a progressive attenuation over the entire 0 to 60 m range, with only localized abrupt declines at some sensing points (e.g., 4 m, 13 m, 16 m, 19 m, and 35 m).

**Figure 7:**
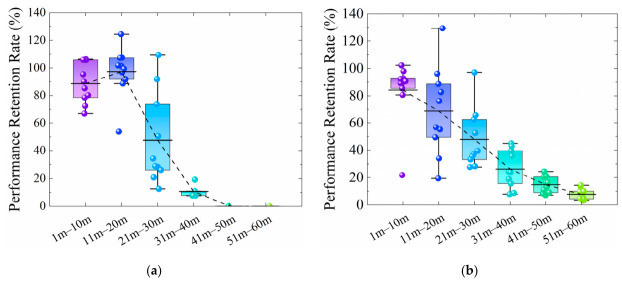
Performance retention rate curves of FBG sensors under the wound condition following integration with geogrids: (**a**) The unarmored sensor; (**b**) The armored sensor.

The performance retention rate curves along the length of the two integrated sensors in the wound condition are presented in Figure 7. For the unarmored FBG sensor, the average retention rates from 1 to 10 m and 11 to 20 m are 88.65% and 97.26%, respectively. A marked decline is observed from 21 to 30 m, with an average value of 47.56%. Conversely, the armored FBG sensor exhibits a nearly linear negative correlation between its performance retention rate and the measurement distance. Quantitatively, the average retention rate decreases at a rate of 18.19% per 10 m within the 1 to 30 m segment, while the decline rate moderates to 9.25% per 10 m over the 31 to 60 m segment.

The gradual attenuation or complete loss of optical signal intensity during propagation in FBG sensors can be attributed to absorption loss, scattering loss, connection loss, and bending loss [29]. In this study, theoretical analysis suggests that bending loss is likely the primary cause of the observed signal attenuation. Bending loss comprises microbending loss (from slight perturbations in the fiber axis) and macrobending loss (from large-scale curvature). During the winding of the FBG-geogrid, the sensors are forced into multiple looped bending structures. Each turn of the sensor is in close contact and under mutual extrusion from adjacent turns and the geogrid itself. A size effect—where the FBG sensor diameter exceeds the yarn thickness—exacerbates this inter-turn extrusion.

Ideally, in a straight FBG sensor, optical energy follows a Gaussian distribution radially, peaking at the fiber axis and decaying to near zero at the cladding boundary [30]. In a bent optical fiber, the phase front of the guided mode must rotate around the center of curvature. This requires an increased phase velocity in the outer region of the bend. When the bending radius falls below a critical value, this phase velocity can exceed that of a plane wave in the cladding, causing some guided modes to convert to radiation modes [31]. This shifts the intensity peak towards the outer core region, leading to radiative leakage and a significant increase in transmission loss.

#### 2.3.2. Performance Under the Flattened Condition

**Figure 8:**
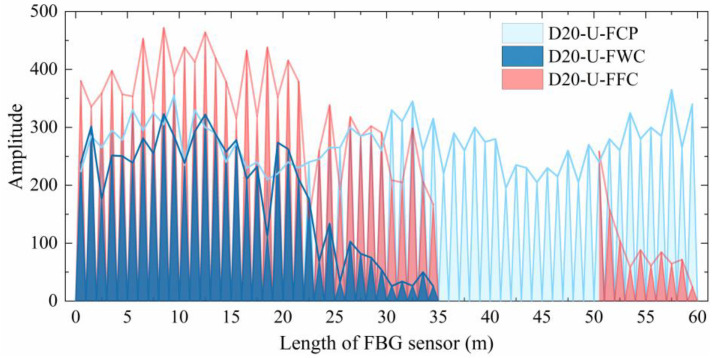
Performance comparison of unarmored FBG sensors under different conditions.

**Figure 9:**
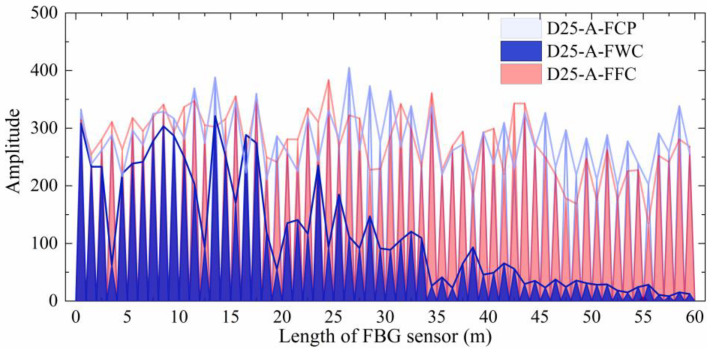
Performance comparison of armored FBG sensors under different conditions.

Figure 8 and Figure 9 compare the optical signal transmission performance of the two integrated FBG sensors under wound and flattened conditions against their initial performance. For D20-U-FFC, effective optical transmission is confined to two segments (0 to 35 m and 50 to 60 m), with at least two complete signal failure points. While its signal intensity in the 0 to 35 m range is generally comparable to or even exceeds that of D20-U-FCP, a marked decline occurs from 50 to 60 m. For D25-A-FFC, effective transmission covers the full 60-m length, and its signal intensity remains essentially consistent with D25-A-FCP.

**Figure 10:**
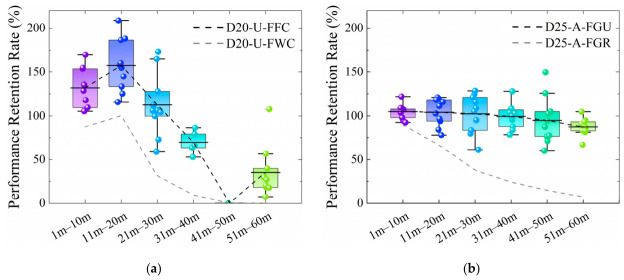
Performance retention rate curves of FBG sensors under different conditions: (**a**) The unarmored sensor; (**b**) The armored sensor.

The corresponding performance retention rate curves are shown in Figure 10. The rate for D20-U-FFC shows an initial increase followed by a decrease. Notably, from 0 to 20 m, its retention rate is significantly higher than that in the wound condition and even exceeds 100% compared to the baseline. The average retention rates are 131.66% (0 to 10 m), 156.33% (11 to 20 m), and 106.05% (21 to 30 m). However, the average plummets to 66.09% from 31 to 40 m, indicating the unstable performance of the unarmored sensor. In contrast, D25-A-FFC demonstrates satisfactory and stable performance, with an average retention rate of 98.86% over 0 to 60 m and a gradual decrease rate of only 2.92% per 10 m. These results prove that the helical steel armor effectively protects the FBG sensor from damage during geogrid manufacturing. This finding aligns with the research of Liao et al. [32], which confirmed that armoring enhances the survival rate of FBG sensors with a negligible impact on their sensitivity.

The observed performance variations can be attributed to several factors. First, upon flattening, the FBG sensors are straightened. The inter-turn extrusion is eliminated, and the bending radius increases, thereby minimizing bending loss. Second, the geogrid production process induces irreversible structural damage (e.g., shear deformation) in sections of the unarmored sensor, leading to severe signal decline or interruption in those regions. Finally, residual tensile strain introduced during manufacturing may reduce optical scattering loss locally, occasionally resulting in a measured performance retention rate slightly above 100%.

#### 2.3.3. Physical Integrity of the Integrated FBG Sensors

**Figure 11:**
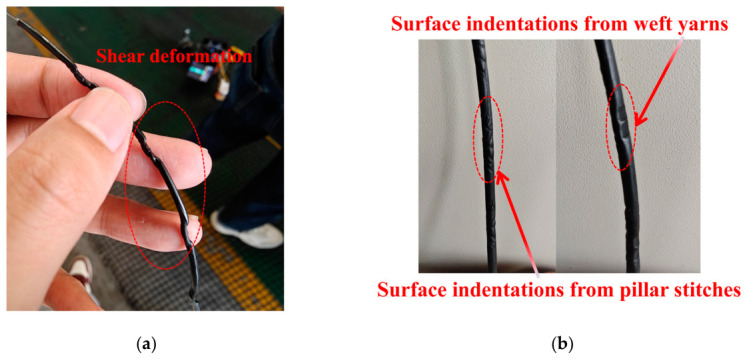
Physical integrity of the FBG sensors separated from FBG-geogrids: (**a**) The unarmored sensor; (**b**) The armored sensor.

The unarmored and armored FBG sensors were separated from the geogrids after performance evaluation. Their physical conditions are shown in Figure 11. Visible shear deformation at the warp-weft yarn intersections was observed on the unarmored FBG sensor. The armored FBG sensor showed no significant shear deformation but displayed distinct surface indentations caused by the weft yarns and pillar stitches.

Considering the production process, material geometry, and physical changes before and after integration, theoretical analysis suggests that the damage to the FBG sensors’ integrity can be described as follows. First, the polyurethane encapsulation layer was softened during the high-temperature drying process at 130 °C. Second, the diameter of the FBG sensors exceeds the thickness of the geogrid yarns, subjecting them to lateral pressure at the yarn intersections. Finally, the transverse and longitudinal stretching of the FBG-geogrid production amplified the shear stress on the sensors at these intersections. The unarmored FBG sensor, being less protected, consequently exhibits significant shear deformation.

### 2.4. Strain Transfer Between the Armored FBG Sensor and Geogrid

The working principle of an FBG sensor is based on the wavelength shift induced by temperature variations and mechanical strain. By monitoring this wavelength drift, strain and temperature can be measured with high precision. Assuming temperature compensation is applied, the sensor responds primarily to axial stress, and the wavelength shift ($\Delta \lambda_{BS}$) can be expressed as follows [18]:

(4) $$\Delta \lambda_{BS}=\left\{1-\frac{\eta_{eff}^{2}}{2}\left[P_{12}-\nu (P_{11}+P_{12})\right]\right\}\times \lambda_{B}\times \Delta \varepsilon =(1-P_{a})\times \lambda_{B}\times \Delta \varepsilon ,$$

where $\lambda_{B}$ is the initial center wavelength, $\eta_{eff}$ is the effective refraction coefficient, ν is Poisson’s ratio, $P_{11}$ and $P_{12}$ are the photo-elastic coefficient, $P_{a}$ is the effective photo-elastic coefficient, and Δε is the axial strain of the FBG sensor.

If $K_{\varepsilon }=\left(1-P_{a}\right)\lambda_{B}$ is the strain sensitivity coefficient of the FBG sensor, then Equation (4) corresponds to the following:

(5) $$\Delta \varepsilon =\frac{1}{K_{\varepsilon }}\Delta \lambda_{BS},$$

The strain sensitivity coefficient ($K_{\varepsilon }$), which quantifies the relationship between strain and wavelength shift, was determined via a calibration test on a universal testing machine, in reference to earlier work [17]. The procedure was as follows: (1) The FBG-geogrid specimen was secured using mechanical grips; (2) The optical fiber was connected to an interrogator; (3) A small pre-tension was applied to ensure a taut state; (4) The machine was set to a constant displacement rate of 0.2 mm/min; (5) During the tensile test, the applied strain on the geogrid and the central wavelength from the FBG sensor were recorded simultaneously. Five parallel tests were conducted.

**Figure 12:**
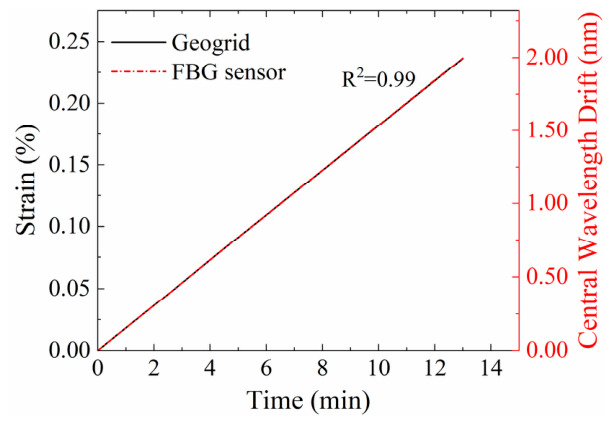
Calibration result through tensile experiments.

The calibration results are presented in Figure 12. A linear relationship is observed between the applied strain on the FBG-geogrid and the central wavelength shift of the FBG sensor. The strain sensitivity coefficient was subsequently calculated based on Equations (4) and (5), $\frac{1}{K_{\varepsilon }}=845\;\mu \varepsilon /nm$.

## 3. Full-Scale Field FBG Sensor Damage Test

The FBG-geogrid was developed to monitor geogrid deformation within asphalt pavements and to elucidate the reinforcement mechanism of geogrid-reinforced structures. Consequently, the integrated FBG sensors must withstand multiple harsh conditions during construction, such as high-temperature asphalt mixtures, roller compaction, and contact with sharp aggregate edges [33]. Therefore, preventing sensor damage during pavement construction is crucial for ensuring their long-term performance and the accuracy of the acquired monitoring data.

### 3.1. Field Test Site

**Figure 13:**
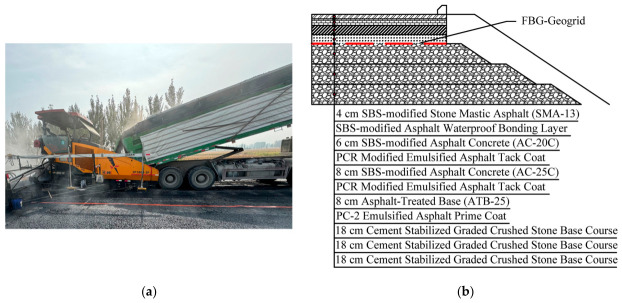
The project Qu-Gang Expressway: (**a**) Pavement structure construction; (**b**) Pavement structure schematic cross-section.

The short-term construction response full-scale field damage test was conducted on the Qu-Gang Expressway project in Hebei Province, China. The pavement structure is illustrated in Figure 13. For this test, the FBG sensors were placed at the bottom of the 8-cm asphalt-treated base (ATB-25) layer.

### 3.2. Physical and Mechanical Properties of Protective Materials

Previous studies have reported the use of materials such as flexible asphalt mastic [34], fine-graded asphalt mixtures [35,36], and silicone rubber [37] for protecting FBG sensors. Based on a comprehensive assessment of protective functionality and on-site construction efficiency, fiberglass-reinforced silicone rubber sheets (FG-SRSs), silicone rubber sheets (SRSs), and rubber sheets (RSs) were preliminarily selected for this field test. All three materials had a thickness of 3 mm.

#### 3.2.1. Thermal Insulation Performance

**Figure 14:**
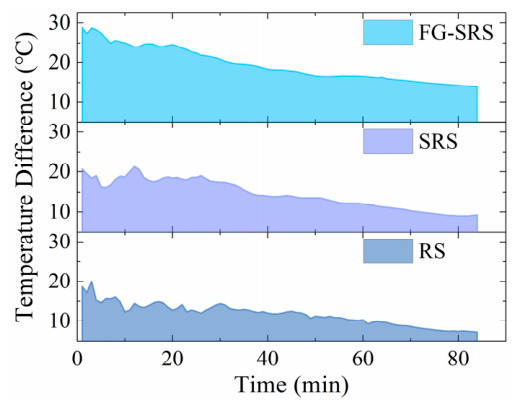
Temperature–time curves of the protective materials.

The thermal insulation performance of the three materials was evaluated in accordance with GB/T 10294-2008 (Thermal Insulation—Determination of Steady-State Thermal Resistance and Related Properties—Guarded Hot Plate Apparatus) [38]. The temperatures of the hot and cold surfaces of the specimens were recorded during testing. The resulting temperature–time curves are plotted in Figure 14. The results indicate that the fiberglass-reinforced silicone rubber sheets (FG-SRSs) exhibit the most superior thermal insulation performance.

#### 3.2.2. Mechanical Puncture Strength

**Figure 15:**
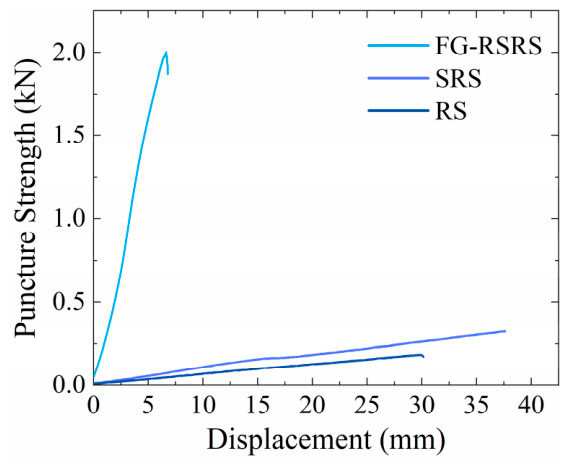
Puncture strength–displacement curves of the protective materials.

Puncture strength tests were conducted in accordance with GB/T 19978-2005 (Geotextiles and Geotextile-Related Products—Determination of Puncture Strength) [39]. The measured puncture strengths of the three materials are shown in Figure 15, following the order: FG-SRS > SRS > RS, with corresponding values of 2.0 kN, 0.7 kN, and 0.4 kN, respectively. Owing to its internal fiber-reinforced layer, the FG-SRS demonstrated the highest puncture strength and exhibited significantly less deformation compared to the SRS and RS.

### 3.3. Equipment and Machinery

The following equipment and machinery were employed, consistent with the Technical Specifications for Construction of Highway Asphalt Pavements (JTG F40-2004) [40]: (1) A cabinet-type distributed optical fiber interrogator was used to monitor the FBG sensors. Its specific parameters are listed in Table 1; (2) A tandem roller with an operating weight of approximately 13 t, vibration frequencies of 40/50 Hz, amplitudes of 0.69/0.29 mm, and excitation forces of 126/84 kN; (3) A pneumatic tire roller with an operating mass of about 30.3 t, a single-tire load of about 3.3 t, and a ground contact pressure of about 545 kPa.

### 3.4. Test Procedure

**Figure 16:**
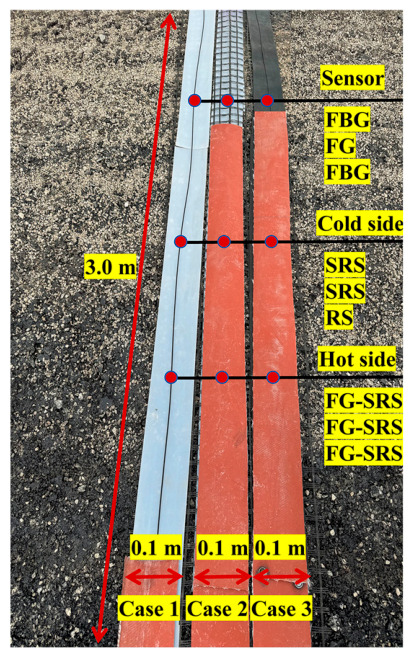
Schematic of the full-scale field FBG sensor damage test. Note: FBG is the armored Fiber Bragg Grating sensor; FG is the FBG-geogrid; Cold side is the surface of cement-stabilized aggregate base; SRS denotes the silicone rubber sheets; RS denotes the rubber sheets; Hot side is the bottom of ATB-25 layer; and FG-SRS denotes fiberglass-reinforced silicone rubber sheets.

Three test conditions were designed for this field test, with specific configurations detailed in Figure 16. Both the protective materials and the armored FBG sensors used were 3 m in length. Each armored FBG sensor contained two monitoring points along its length, designated as Point 1 and Point 2. It should be noted that for each of the three cases, parallel tests were carried out—one comprised normal asphalt paving and compaction, and the other involved paving only for temperature compensation. Every test was repeated three times. The asphalt mixture and construction machinery strictly followed the actual project specifications to ensure the validity and practical relevance of the test results.

**Figure 17:**
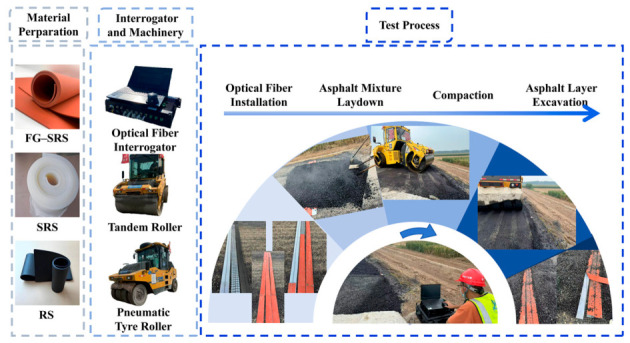
Test procedure of the full-scale field FBG sensors damage test.

The full-scale field test consisted of four consecutive stages: installation of protective materials and FBG sensors, asphalt mixture paving, compaction, and a stationary monitoring phase. The compaction scheme is presented in Table 2, and the overall test procedure is illustrated in Figure 17.

### 3.5. Test Results

**Figure 18:**
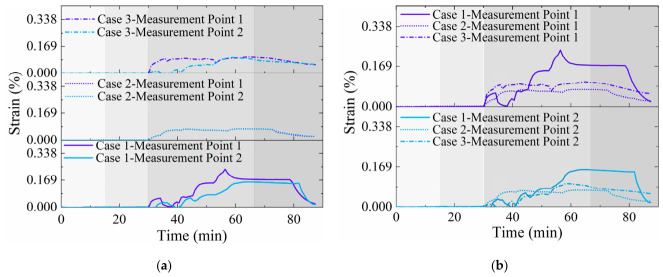
Strain–time curves for the three test cases: (**a**) Results from different points along one FBG sensor; (**b**) Results at the same point from different FBG sensors.

Figure 18 shows the strain–time curves of the FBG sensors recorded during asphalt pavement construction. All curves exhibit a characteristic three-stage sequence: initial stress accumulation, peak stabilization, and subsequent unloading/rebound. This response corresponds precisely to the application and removal of high-temperature and mechanical loads during construction, confirming that the protective materials effectively shielded the FBG sensors. However, distinct differences are evident among the strain curves obtained with different protective materials. A detailed analysis is provided below.

Figure 18a presents strain results from different measurement points along the same FBG sensor (same test case). The following observations can be made: (1) In Case 2 and Case 3, the strain curves vary relatively smoothly without abrupt changes, whereas those for Case 1 show significant fluctuations. (2) The FBG sensor in Case 2 demonstrates the most stable response, with minimal strain variation between its measurement points; Case 3 follows in stability, and Case 1 exhibits the least stability.

Figure 18b illustrates the strain variation at the same monitoring point (Point 1) across the three different test conditions. Key observations are as follows: (1) The FBG sensor in Case 1 exhibits the highest peak strain (0.238%), while the peaks in Case 2 and Case 3 are 0.071% and 0.104%, respectively. (2) At the end of the test, the residual strain in Case 3 remains the largest (approximately 0.058%), whereas the residual strains in Case 1 and Case 2 are only 0.020% and 0.025%, respectively.

Note: The background shading, from light to dark, represents the sequential stages: sensor installation, asphalt paving, compaction, and the stationary phase.

**Figure 19:**
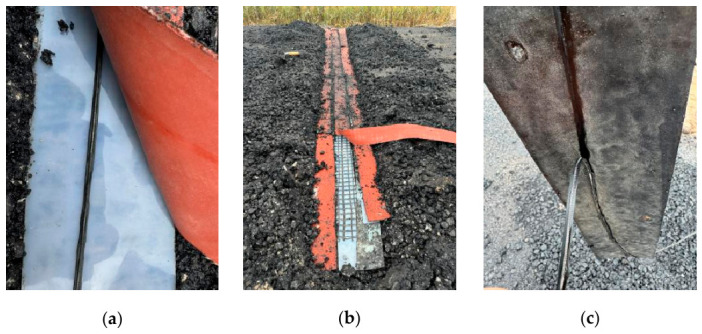
Post-test physical integrity of FBG sensors: (**a**) Case 1; (**b**) Case 2; (**c**) Case 3.

Figure 19 shows the physical condition of the FBG sensors after removal of the overlying asphalt mixture. Under the combined high-temperature and compaction loads, the polyurethane encapsulation layer softened in all cases, compromising its protection of the optical fiber core. Indentations are visible on the silicone rubber and rubber sheets in all cases. Notably, in Case 3, the FBG sensor became embedded and adhered to the rubber sheet. These findings underscore that the helical steel armor is an essential structural component for the FBG sensor’s mechanical integrity.

Based on the strain monitoring results (Figure 18) and the post-test physical inspection (Figure 19), it can be concluded that the silicone rubber sheet placed beneath the FBG sensor (cold side) introduces minimal interference with the strain response. In contrast, the rubber sheet exhibited significant softening at high temperatures due to its inferior heat resistance, which substantially altered and interfered with the sensor’s deformation response.

## 4. Conclusions

To address the challenge of monitoring the mechanical response of geogrids in asphalt pavements using Fiber Bragg Grating (FBG) sensors, this study first evaluated the performance of two types of FBG sensors integrated with geogrids via an industrial warp-knitting process. A full-scale field damage test was subsequently conducted on an expressway project. The main findings are summarized as follows:

(1) The unarmored FBG sensor fractured during industrial production of the FBG-geogrid under the combined effects of high-temperature drying and shear stresses from longitudinal and transverse stretching. Consequently, its effective optical signal transmission range degraded from an initial continuous 60 m to two discontinuous segments (0 to 35 m and 50 to 60 m), confirming its incompatibility with this manufacturing process.

(2) Although the armored FBG sensor (with a helical steel layer) maintained a complete optical transmission range (0 to 60 m), its performance was compromised in the wound configuration. The winding process induced significant circular bending, with each turn under tight contact and compression, leading to a progressive decline in the performance retention rate of approximately 15.29% per 10 m. This resulted in an average retention of only 41.56% over the full 60-m length.

(3) When the FBG-geogrid was flattened, bending losses in the armored sensor were effectively eliminated. Additionally, residual tensile strain within the sensor reduced scattering losses, leading to a significant performance recovery and an average retention rate of 98.86% over the 0 to 60 m range.

(4) A strong linear correlation was established between the wavelength shift of the armored FBG sensor and the tensile strain of the geogrid. This correlation is attributed to the binding effect of the pillar stitches, which integrate the sensor and geogrid into a cohesive unit and ensure coordinated deformation.

(5) The full-scale field test demonstrated that a protection scheme employing silicone rubber on the cooler side and fiberglass-reinforced silicone rubber on the hotter side effectively shielded the FBG-geogrid from short-term construction damage. Conversely, materials with inferior heat resistance, such as standard rubber, are not recommended.

In summary, the developed FBG-geogrid establishes a robust sensor-host system, providing a reliable means to monitoring the performance of asphalt pavements. Considering the thickness and in-pavement interaction of the protective layers, further optimization of both the FBG-geogrid and its protection scheme may be necessary for applications in intermediate and surface pavement layers, even though the protective materials themselves are small in size.

## Figures and Tables

**Table 1:** Parameters of the optical fiber interrogator.

Parameters	Values
number of channels (unit)	16
wavelength range (nm)	1528~1568
wavelength resolution (pm)	1
repeatability (pm)	±5
demodulation rate (Hz)	≥1
dynamic range (dB)	35
optical interface type	FC/APC

**Table 2:** Compaction scheme.

Stage	Roller Type	Speed (km/h)	Temperature (°C)	Number of Passes
Initial compaction	Tandem roller	2–3	≥135	2
Intermediate compaction	Pneumatic tire roller	3–5	≥125	3
Final compaction	Tandem roller	3–4.5	≥90	2

Note: The initial compaction consisted of two passes: the first with the front drum static and rear drum vibrating, followed by a pass with both drums vibrating.

## Data Availability

The original contributions presented in this study are included in the article. Further inquiries can be directed to the corresponding author.

## Abbreviations

The following abbreviations are used in this manuscript:

FBG	Fiber Bragg Grating
TDM	Time-division multiplexing
OFDR	Optical frequency domain reflectometry
FCP	Fiberglass commercially packaged
FWC	Fiberglass wound condition
FFC	Fiberglass flattened condition
D	Diameter
U	Unarmored
A	Armored
ATB	Asphalt-treated base
FG-SRSs	Fiberglass-reinforced silicone rubber sheets
SRSs	Silicone rubber sheets
RSs	Rubber sheets
